# Analysis of risk factors and construction of nomogram model for cardiac valve calcification of patients undergoing hemodialysis

**DOI:** 10.3389/fcvm.2026.1675197

**Published:** 2026-02-19

**Authors:** Yan Zhang, Lin Huang, Mengjun Tao, Jiajun Zhou, Deguang Wang

**Affiliations:** 1 Department of Nephrology, The Second Affiliated Hospital of Anhui Medical University, Hefei, Anhui, China; 2 Blood Purification Center, Affiliated Yijishan Hospital of Wannan Medical College, Wuhu, Anhui, China; 3 Department of Health Management Center, First Affiliated Hospital of Wannan Medical College, Wuhu, Anhui, China

**Keywords:** cardiac valve calcification, fat tissue index, hemodialysis, nomogram, risk factors

## Abstract

**Objective:**

This study aimed to construct a risk prediction nomogram model of cardiac valve calcification (CVC) in patients undergoing maintenance hemodialysis (MHD) and to verify its evaluation effect.

**Methods:**

A total of 398 patients undergoing hemodialysis were randomly divided into a modeling group (*n* = 274) and a validation group (*n* = 124). In the modeling group, 92 patients had CVC and 182 did not. Multivariate logistic regression analysis was conducted to determine the risk factors for CVC in patients undergoing hemodialysis. A nomogram prediction model was constructed using R software, and its predictive performance was evaluated in terms of discrimination, calibration, and clinical utility.

**Results:**

This study included 398 patients undergoing MHD with a mean age of 51.17 ± 14.09 years, and the prevalence of CVC was 31.66%. Compared with the non-CVC group, patients in the CVC group were older and had a higher proportion of males, longer dialysis duration, higher prevalence of diabetes, and higher levels of total cholesterol, triglycerides, and fat tissue index, while handgrip strength was significantly lower (all *P* < 0.05). Multivariate logistic regression analysis identified age (OR = 1.052, 95%*CI* = 1.028–1.077), male sex (OR = 3.164, 95%*CI* = 1.679–5.962), dialysis duration ≥ 36 months (OR = 2.096, 95%*CI* = 1.162–3.781), total cholesterol level (OR = 1.582, 95%*CI* = 1.191–2.101), and fat tissue index (OR = 1.128, 95%*CI* = 1.046–1.217) as independent risk factors for CVC (all *P* < 0.05). The area under the receiver operating characteristic curve (AUC) of the nomogram in the modeling group was 0.789, indicating good discriminative ability. The calibration curve demonstrated good agreement between predicted and observed outcomes. In the validation group, the AUC was 0.751, with calibration curve closely aligned with the ideal reference line. Decision curve analysis (DCA) further confirmed the clinical utility of the nomogram.

**Conclusion:**

Patients undergoing hemodialysis who are older, male, have a dialysis duration ≥ 36 months, elevated total cholesterol levels, and increased fat tissue index are at higher risk of developing CVC. The nomogram model demonstrated good predictive performance for CVC in patients undergoing hemodialysis and may serve as a practical tool for identifying high-risk individuals in clinical practice.

## Introduction

Hemodialysis (HD) is the mainstay of renal replacement therapy in patients with end-stage renal disease (ESRD). Cardiovascular disease (CVD) is a common complication in patients undergoing maintenance hemodialysis (MHD) and remains the leading cause of death in this population (1). Meta-analyses have shown that dialysis patients with cardiac valve calcification (CVC) have a 1.31-fold increased risk of cardiovascular mortality compared with those without CVC (2). CVC not only contributes to cardiovascular complications such as valvular insufficiency or stenosis, heart failure, myocardial infarction, and conduction abnormalities, but is also closely associated with impaired quality of life and reduced long-term survival. Moreover, CVC is a strong predictor of cardiovascular events and all-cause mortality in dialysis patients (3).

Although vascular calcification (VC) in hemodialysis patients has received increasing attention in recent years, CVC remains a major clinical challenge due to its poor reversibility and substantial impact on outcomes. Patients at higher risk of CVC may require closer surveillance because of factors such as advanced age, disturbances in calcium-phosphorus metabolism, and comorbid conditions. While numerous clinical studies have explored the factors associated with CVC in patients undergoing hemodialysis, the determinants of CVC in this population have not yet been fully elucidated.

Handgrip strength (HGS) has been proposed as a simple and reliable indicator of overall muscle strength. Functional decline is thought to precede, and may even exceed, the loss of muscle mass during ageing (4). Previous studies have demonstrated that lower muscle strength, as assessed by HGS, is associated with higher abdominal aortic calcification (AAC) scores in adults aged ≥40 years in the United States (5). Bioelectrical impedance analysis (BIA), which is based on the electrical properties of biological tissue, has been widely used to evaluate body composition (6). BIA plays an important role in the diagnosis of uremic sarcopenia, assessment of nutritional status, and determination of the relationship between nutritional status and VC (7–9). However, the association between CVC and body composition indicators in patients undergoing hemodialysis remains unclear.

Therefore, this study enrolled 398 patients undergoing hemodialysis to identify risk factors associated with cardiac valve calcification. In addition, a risk prediction model was developed to facilitate early identification of high-risk individuals and to provide a basis for future targeted interventions.

## Data and methods

### General data

A single-center cross-sectional analysis was performed. A total of 398 patients undergoing maintenance hemodialysis at our hospital between December 2021 and December 2024 were enrolled and randomly divided into a modeling group (*n* = 274) and a validation group (*n* = 124). Within the modeling group, patients were further categorized into CVC group (*n* **=** 92) and non-CVC group (*n* = 182) according to the presence or absence of cardiac valve calcification, as determined by echocardiographic examination. This study was approved by the clinical ethics committee of our hospital and met ethical standards. The ethics committee number was 021 (2021). Patients who refused to provide written consent were excluded from the study.

The inclusion criteria were as follows: ① age >18 years; ② End-stage renal disease; maintenance hemodialysis should not be less than 3 months; ③ patients received dialysis three times a week. Exclusion criteria were: ① patients with confirmed rheumatic valvular heart disease; ② patients with congenital heart disease; ③ presence of infective endocarditis by cardiac color ultrasound.

### Data collection

Clinical data were collected from patients undergoing maintenance hemodialysis, including age, sex, primary kidney disease, duration of hemodialysis, presence or absence of diabetes mellitus, pre-dialysis systolic blood pressure (Pre-SBP), and pre-dialysis diastolic blood pressure (Pre-DBP). Laboratory parameters were obtained from blood samples collected every three month during the study period, and mean values were calculated. These parameters included hemoglobin (Hb), blood urea nitrogen (BUN), serum creatinine (Scr), serum albumin (Alb), total cholesterol (Tcho), triglyceride (TG), serum calcium (Ca), serum phosphate (P), serum potassium (K), and intact parathyroid hormone (iPTH). Single-pool Kt/v (spKt/V) was calculated using the following formula: K⋅t/V=−ln⁡(R−0.008⋅t)+(4–3.5⋅R)ΔBW/BW, where R represents the ratio of post-dialysis to pre-dialysis blood urea nitrogen, t is the dialysis duration (hours), BW is body weight, ΔBW indicates the change in body weight before and after hemodialysis.

### Body composition and handgrip strength measurements

Multi-frequency bioelectrical impedance analysis was performed using a Body Composition Monitor (BCM, Fresenius Medical Care, Germany). Measurements were obtained following a 10-minute rest period in the supine position before dialysis. Electrodes were placed on the patient's wrist and ankle within a distance of 5 cm on the contralateral side of the arteriovenous fistula. We gathered information on the lean tissue index (LTI), fat tissue index (FTI), LTI = lean tissue mass/height^2^ (kg/m^2^), and FTI = adipose tissue mass/height^2^ (kg/m^2^). Both LTI and FTI are internally calculated by the BCM and are theoretically unaffected by hydration status (10). HGS was assessed in the hand of the limb without an arteriovenous fistula, using an electronic grip dynamometer (ZhongYiheng Corporation, Hangzhou, China). Patients were instructed to apply maximum force after the voice command. The measurement was performed three times at 1 min intervals. For the analysis, the highest value obtained was considered. Low muscle strength was defined as handgrip strength (HGS) <18 kg in women and <28 kg in men (11).

### Calcification of cardiac valves diagnosed by echocardiography

All patients underwent transthoracic echocardiography (TTE), which was performed by experienced echocardiographers using a standardized ultrasound system. During the examination, patients were positioned in the left lateral decubitus position, and images were acquired according to standard views recommended by the American Society of Echocardiography. Valvular calcification was defined as the presence of increased echogenicity with dense, irregular hyperechoic structures at the valve leaflets or annulus, with or without acoustic shadowing, and persisting across multiple imaging planes. The diagnostic criteria for CVC are the presence of high echogenic signals greater than 1 mm on the aortic valve, mitral valve, or mitral annulus (12, 13). To minimize observer bias, all echocardiographic images were independently interpreted by two blinded echocardiographers, and discrepancies were resolved by a third senior physician.

### Statistical processing

SPSS 25.0 (IBM Corp., NY, USA) was used for the analysis. The Kolmogorov–Smirnov test was used for normality analysis of the quantitative variables. Continuous variables conforming to normal distribution were represented as mean ± standard deviation, independent *t*-tests were performed between the groups, and non-normally distributed continuous variables were presented as medians and interquartile ranges and compared using the Mann–Whitney U test. The count data were expressed as numbers and percentages, and the chi-square (*χ*^2^) test was performed between the groups. Multiple logistic regression analysis was performed to determine the risk factors for CVC development in patients undergoing hemodialysis. R software (R4.4) and the *rms* program package were used to construct a nomogram model for predicting the risk of CVC in hemodialysis patients. The receiver operating characteristic (ROC) curve and the area under the curve (AUC) were calculated to evaluate the predictive value and discrimination of the nomogram model. A calibration curve was plotted to evaluate the predictive accuracy of the nomogram model. Decision curve analysis (DCA) was also used to assess the clinical utility of the model. *P* < 0.05 indicates statistically significant difference.

## Results

### Demographic data

**Figure 1:**
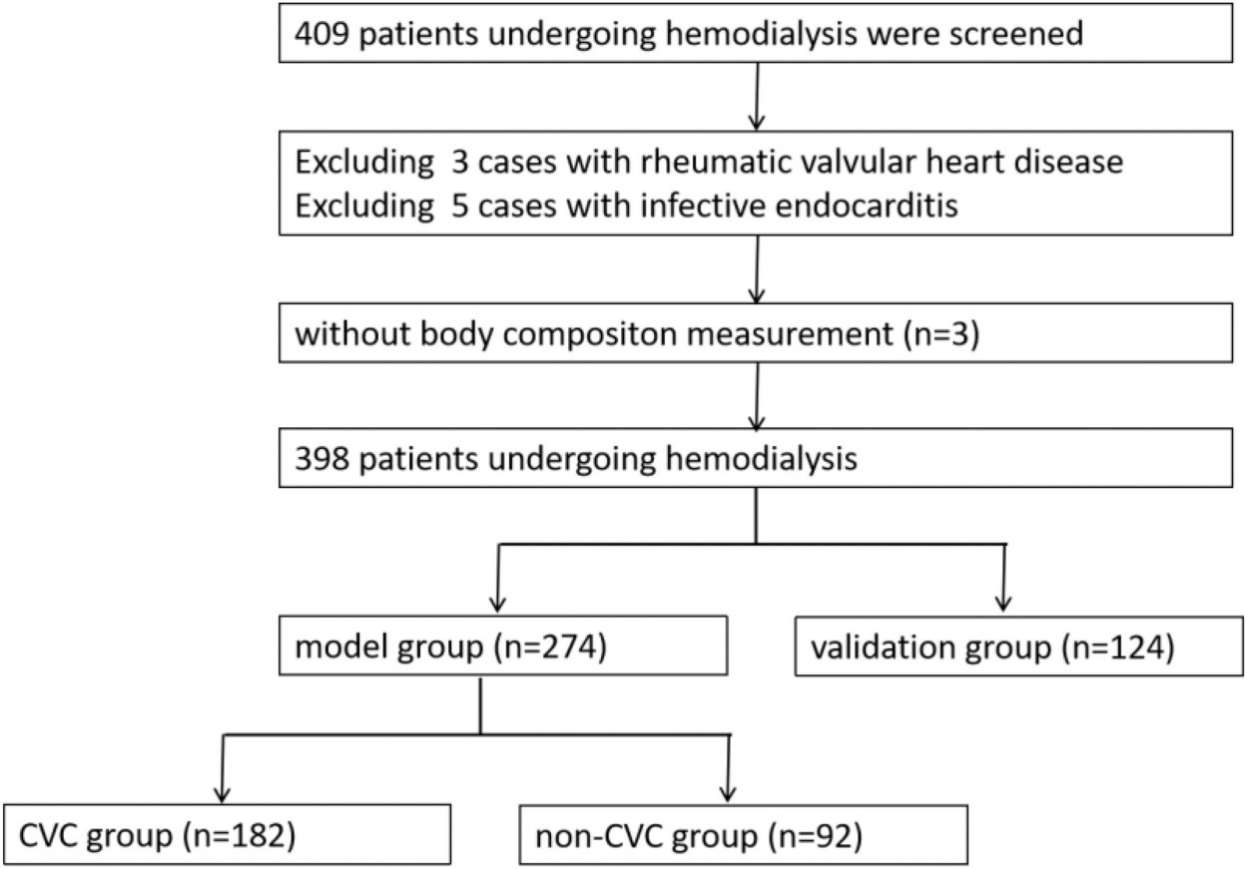
Flow chart. CVC, cardiac valve calcification.

Our study included 398 patients undergoing maintenance hemodialysis (MHD) (Figure 1), of whom 225 were males (56.53%). The mean age of the study population was 51.17 ± 14.09 years. The median duration of hemodialysis was 39 months (interquartile range: 18.75, 64 months), and 216 patients (54.27%) had a hemodialysis duration of ≥36 months. The underlying causes of end-stage renal disease (ESRD) included chronic glomerulonephritis in 142 patients (35.68%), diabetic nephropathy in 124 patients (31.16%), hypertensive nephropathy in 86 patients (21.61%), interstitial nephritis in 10 patients (2.51%), polycystic kidney disease in 9 patients (2.26%), and other etiology in 27 patients (6.78%). A total of 130 patients (32.66%) had concomitant diabetes mellitus. Low handgrip strength (HGS) was identified in 102 patients (25.63%). Regarding vascular access, 371 patients (93.22%) had an arteriovenous fistula, whereas 27 patients (6.78%) used a central venous catheter. Cardiac valve calcification (CVC) was identified in 126 patients (31.66%). Among these patients, 95 (75.40%) had aortic valve calcification (AVC), 58 (46.03%) had mitral valve calcification (MVC), 27 (21.43%) had both AVC and MVC, and 4 patients (3.17%) had tricuspid valve calcification (TVC). As shown in Table 1.

**Table 1:** Comparison of clinical information between the modeling group and the validation group.

Index	Total cases	Modeling	Validation	* t/Z/χ2*	*P*
	(*n* = 398)	Group (*n* = 274)	Group (*n* = 124)		
Age (years)	51.17 ± 14.09	50.97 ± 13.94	51.59 ± 14.47	0.402	0.688
Sex				0.458	0.498
Male [*n* (%)]	225 (56.53%)	158 (57.66%)	67 (54.03%)		
Female [*n* (%)]	173 (43.47%)	116 (42.34%)	57 (45.97%)		
Dialysis duration (month)	39 (18.75,64)	36 (18, 59.25)	44 (19, 68)	−0.627	0.531
Dialysis duration ≥ 36 months				0.345	0.557
Yes [*n* (%)]	216 (54.27%)	146 (53.28%)	70 (56.45%)		
No [*n* (%)]	182 (45.73%)	128 (46.72%)	54 (43.55%)		
With diabetes				0.332	0.564
Yes [*n* (%)]	130 (32.66%)	87 (32.75%)	43 (34.68%)		
No [*n* (%)]	268 (67.34%)	187 (68.25%)	81 (65.32%)		
Cause of ESRD				3.889	0.566
Chronic glomerulonephritis	142 (35.68%)	95 (34.67%)	47 (37.90%)		
Diabetic nephropathy	124 (31.16%)	83 (30.29%)	41 (33.06%)		
Hypertensive nephropathy	86 (21.61%)	59 (21.53%)	27 (21.77%)		
Interstitial nephritis	10 (2.51%)	9 (3.29%)	1 (0.81%)		
Polycystic nephropathy	9 (2.26%)	7 (2.56%)	2 (1.62%)		
Other etiology	27 (6.78%)	21 (7.66%)	6 (4.84%)		
Pre-SBP	149.85 ± 23.66	149.37 ± 23.25	150.92 ± 24.60	0.604	0.546
Pre-DBP	82.82 ± 14.62	82.66 ± 14.95	83.50 ± 13.89	0.529	0.597
Hb	109.41 ± 17.46	109.05 ± 17.62	110.19 ± 17.13	0.599	0.550
BUN	23.86 ± 5.34	23.82 ± 5.39	23.95 ± 5.25	0.237	0.812
Scr	914.88 ± 243.62	918.61 ± 232.90	906.63 ± 266.63	−0.430	0.668
Alb	40.11 ± 3.58	40.05 ± 3.56	40.24 ± 3.63	0.485	0.628
Tcho	4.03 ± 1.03	4.05 ± 1.03	3.99 ± 1.02	−0.548	0.584
TG	1.90 ± 1.49	1.96 ± 1.61	1.75 ± 1.1699	−1.304	0.193
K	4.95 ± 0.78	4.92 ± 0.76	5.04 ± 0.80	1.058	0.291
Ca	2.17 ± 0.21	2.17 ± 0.21	2.17 ± 0.22	−0.097	0.923
P	1.82 ± 0.51	1.81 ± 0.49	1.84 ± 0.55	0.580	0.562
iPTH	385.35 (208.73,678.80)	367.5 (202.53, 638.68)	431.20 (238.38, 781.63)	0.047	0.943
LTI	13.51 ± 2.71	13.58 ± 2.73	13.35 ± 2.68	−0.782	0.435
FTI	9.85 ± 4.30	9.77 ± 4.27	10.03 ± 4.36	0.564	0.573
HGS	28.40 ± 9.58	28.57 ± 9.52	28.08 ± 9.75	−0.478	0.633
Low HGS				0.092	0.762
Yes [*n* (%)]	102 (25.63%)	69 (25.18%)	33 (26.61%)		
No [*n* (%)]	296 (74.37%)	205 (74.82%)	91 (73.39%)		
spKt/V	1.35 ± 0.35	1.36 ± 0.37	1.33 ± 0.30	−0.547	0.585
CVC				1.496	0.221
Yes [*n* (%)]	126 (31.66%)	92 (33.58%)	34 (27.42%)		
No [*n* (%)]	272 (68.34%)	182 (66.42%)	90 (72.58%)		
AVC	95 (75.40%)	66 (71.74%)	29 (85.29%)		
MVC	58 (46.03%)	44 (47.83%)	14 (41.18%)		
AVC + MVC	27 (21.43%)	19 (20.65%)	8 (23.53%)		

CVC, cardiac valve calcification; ESRD, end-stage renal disease; Pre-SBP, pre-dialysis systolic blood pressure; Pre-DBP, predialysis diastolic blood pressure;Hb, hemoglobin; BUN, blood urea nitrogen; Scr, serum creatinine; Alb, albumin; Tcho, total cholesterol; TG, triglyceride; K, serum potassium; Ca, serum calcium; P, serum phosphate; iPTH, intact parathyroid hormone; LTI, lean tissue index; FTI, fat tissue index; HGS, handgrip strength; spKt/V, single-pool Kt/V; AVC, aortic valve calcification; MVC, mitral valve calcification.

### Comparison of clinical data between the modeling and the validation groups

There were no statistically significant differences between the modeling group and the validation group with respect to age, sex, dialysis duration, dialysis duration ≥36 months, presence of diabetes mellitus, cause of ESRD, pre-dialysis systolic blood pressure (Pre-SBP), pre-dialysis diastolic blood pressure (Pre-DBP), hemoglobin (Hb), blood urea nitrogen (BUN), serum creatinine (Scr), serum albumin (Alb), total cholesterol (Tcho), triglycerides (TG), serum potassium (K), serum calcium (Ca), serum phosphate (P), intact parathyroid hormone (iPTH), lean tissue index (LTI), fat tissue index (FTI), handgrip strength (HGS), Low HGS, single-pool Kt/V (spKt/V), and cardiac valve calcification (CVC) (all *P* > 0.05), as shown in Table 1.

### Comparison of clinical data between CVC and non-CVC patients in the modeling group

There were no statistically significant differences between the CVC and non-CVC groups with respect to pre-dialysis systolic blood pressure (Pre-SBP), pre-dialysis diastolic blood pressure (Pre-DBP), hemoglobin (Hb), blood urea nitrogen (BUN), serum creatinine (Scr), serum albumin (Alb), serum potassium (K), serum calcium (Ca), serum phosphate (P), intact parathyroid hormone (iPTH), lean tissue index (LTI) or spKt/V (all *P* > 0.05). In contrast, age, male sex, dialysis duration, dialysis duration ≥36 months, presence of diabetes mellitus, total cholesterol (Tcho), triglycerides (TG), and fat tissue index (FTI) were significantly higher in the CVC group than in the non-CVC group, whereas handgrip strength (HGS) was significantly lower in the CVC group (all *P* < 0.05), as shown in Table 2.

**Table 2:** Comparison of clinical data between CVC and non-CVC patients in the modeling group.

Index	Non-CVC	CVC	* t/Z/χ2*	*P*
	Group (*n* = 182)	Group (*n* = 92)		
Age (years)	47.55 ± 13.64	57.74 ± 11.96	−6.077	0.000
Sex			4.235	0.040
Male [*n* (%)]	97 (53.30%)	61 (66.30%)		
Female [*n* (%)]	85 (46.70%)	31 (33.70%)		
Dialysis duration (month)	35 (18, 57)	46 (21.5, 65)	2.017	0.044
Dialysis duration ≥ 36 months			4.184	0.041
Yes [*n* (%)]	89 (48.90%)	57 (61.96%)		
No [*n* (%)]	93 (51.10%)	35 (38.04%)		
With diabetes			5.832	0.016
Yes [*n* (%)]	49 (26.92%)	38 (41.30%)		
No [*n* (%)]	133 (73.08%)	54 (58.70%)		
Pre-SBP	148.32 ± 23.80	151.42 ± 22.12	−1.043	0.298
Pre-DBP	83.77 ± 15.03	80.47 ± 14.64	1.730	0.085
Hb	108.32 ± 16.87	110.51 ± 19.05	−0.965	0.336
BUN	23.8 ± 4.85	23.85 ± 6.35	−0.077	0.939
Scr	937.87 ± 233.50	880.92 ± 228.29	1.918	0.056
Alb	40.31 ± 3.64	39.55 ± 3.35	1.676	0.095
Tcho	3.91 ± 0.89	4.33 ± 1.22	−2.915	0.004
TG	1.76 ± 1.19	2.36 ± 2.16	−2.485	0.014
K	4.82 ± 0.71	4.89 ± 0.84	−0.747	0.456
Ca	2.173 ± 0.21	2.164 ± 0.20	0.311	0.756
P	1.818 ± 0.49	1.80 ± 0.50	0.338	0.736
iPTH	385.80 (202.30, 602.30)	431.20 (241.10, 870.73)	1.679	0.093
LTI	13.71 ± 2.67	13.32 ± 2.85	1.118	0.264
FTI	9.08 ± 4.03	11.14 ± 4.44	−3.855	0.000
HGS	29.39 ± 9.80	26.87 ± 8.74	2.081	0.038
Low HGS			6.775	0.009
Yes [*n* (%)]	37 (20.33%)	32 (34.78%)		
No [*n* (%)]	145 (79.67%)	60 (65.22%)		
spKt/v	1.35 ± 0.34	1.36 ± 0.428	−0.197	0.844

CVC, cardiac valve calcification; Pre-SBP, pre-dialysis systolic blood pressure; Pre-DBP, pre-dialysis diastolic blood pressure;Hb, hemoglobin; BUN, blood urea nitrogen; Scr, serum creatinine; Alb, albumin; Tcho, total cholesterol; TG, triglyceride; K, serum potassium; Ca, serum calcium; P, serum phosphate; iPTH, intact parathyroid hormone; LTI, lean tissue index; FTI, fat tissue index; HGS, handgrip strength; spKt/v, single-pool Kt/v.

### Multiple logistic regression analysis of CVC in patients undergoing hemodialysis

The presence of cardiac valve calcification (CVC) was used as the dependent variable (1 = presence, 0 = absence). Independent variables included sex (1 = male, 0 = female), dialysis duration ≥ 36 months (1 = yes, 0 = no), diabetes mellitus (1 = yes, 0 = no), low handgrip strength (low HGS; 1 = yes, 0 = no), as well as age, total cholesterol (Tcho), triglycerides (TG), and fat tissue index (FTI), which were treated as continuous variables. These variables were selected for multivariate logistic regression analysis based on their statistical significance in the univariate analysis (Table 3). The results of the multivariate logistic regression analysis demonstrated that age, male sex, dialysis duration ≥36 months, total cholesterol level, and fat tissue index were independent risk factors for CVC in patients undergoing hemodialysis (all *P* < 0.05) (Table 4). The prediction model was expressed as follows: Z = 0.051 × Age + 1.152 × Sex + 0.740 × Dialysis duration ≥36 months + 0.458 × Total cholesterol + 0.120 × Fat tissue index—7.555.

**Table 3:** Univariate logistic regression analysis of the CVC in patients undergoing hemodialysis.

Influence factor	B	OR	95%CI	*P*
Age	0.058	1.060	1.038–1.082	0.000
Male sex	0.545	1.724	1.024–2.904	0.040
Dialysis duration ≥ 36 months	0.532	1.702	1.020–2.838	0.042
With Diabetes	0.647	1.910	1.126–3.241	0.016
Tcho	0.403	1.496	1.157–1.934	0.002
TG	0.239	1.270	1.066–1.514	0.007
FTI	0.115	1.122	1.054–1.194	0.000
Low HGS	0.737	2.090	1.193–3.662	0.010

CVC, cardiac valve calcification; Tcho, total cholesterol; TG, triglyceride; FTI, fat tissue index; HGS, handgrip strength.

**Table 4:** Multiple logistic regression analysis of the CVC in patients undergoing hemodialysis.

Influence factor	B	SE	Wald	OR	95%CI	*P*
Age	0.051	0.012	18.745	1.052	1.028–1.077	0.000
Male	1.152	0.323	12.690	3.164	1.679–5.962	0.000
Dialysis duration ≥ 36 months	0.740	0.301	6.053	2.096	1.162–3.781	0.014
Tcho	0.458	0.145	10.007	1.582	1.191–2.101	0.002
FTI	0.120	0.039	9.721	1.128	1.046–1.217	0.002
Constant	−7.555	1.070	49.853	0.001		0.000

CVC, cardiac valve calcification; Tcho, total cholesterol; FTI, fat tissue index.

### Construction of a risk prediction nomogram model for CVC in patients undergoing hemodialysis

**Figure 2:**
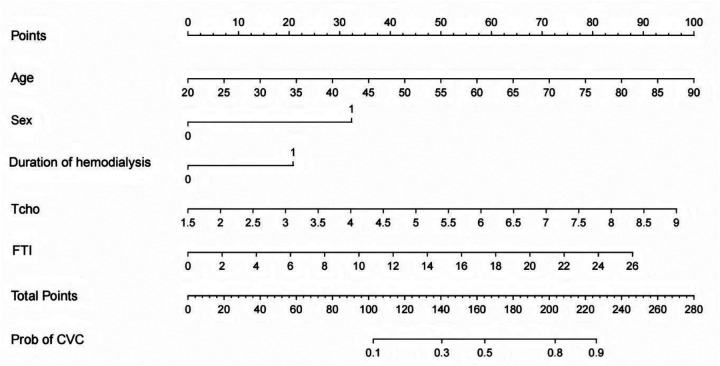
Nomogram model for predicting the risk of CVC in patients undergoing hemodialysis. CVC, cardiac valve calcification; Tcho, total cholesterol; FTI, fat tissue index; Sex, 0: female, 1: male; Duration of hemodialysis, 0: <36months, 1: ≥ 36months.

Based on the results of the multiple logistic regression analysis, a nomogram was constructed to predict the risk of cardiac valve calcification (CVC) in patients undergoing maintenance hemodialysis. The nomogram assigns 7.14 points for every 5-years increase in age, 33.34 points for male sex, 20.79 points for a dialysis duration ≥ 36 months, 6.44 points for every 0.5 mmol/L increase in total cholesterol, and 6.76 points for every 2 kg/m^2^ increase in fat tissue index. A graphical representation of the nomogram is shown in Figure 2.

### Internal validation of the nomogram model

**Figure 3:**
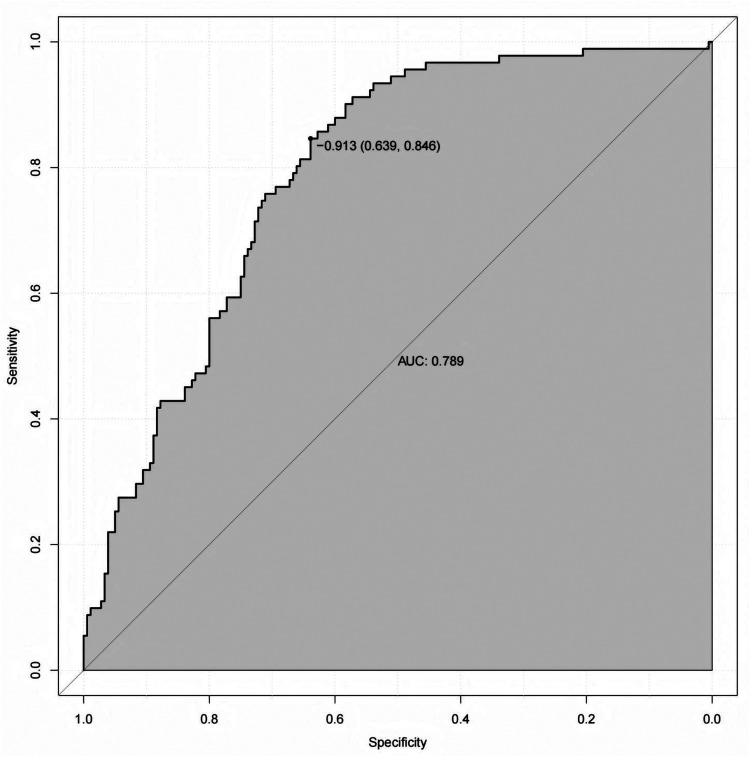
Receiver operating characteristic of the modeling cohort for predicting the CVC in patients undergoing hemodialysis.

**Figure 4:**
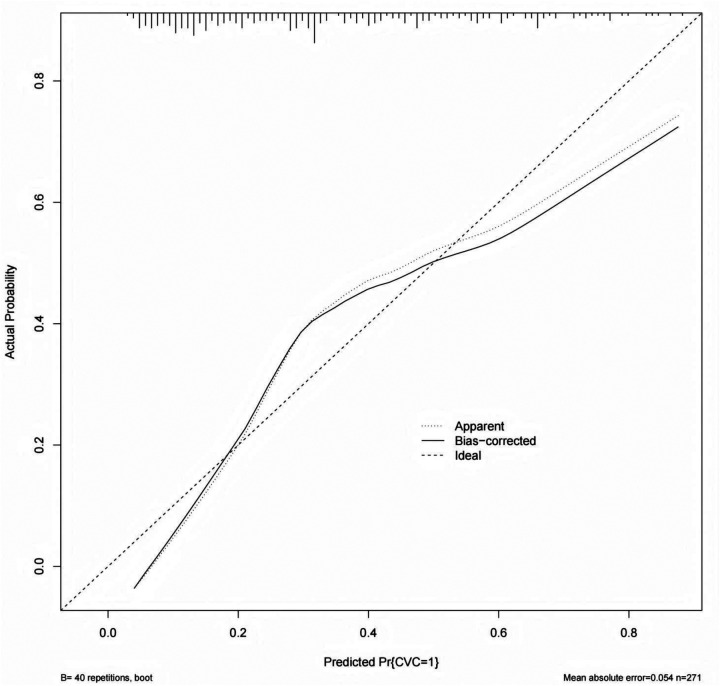
Calibration curve of the modeling cohort for predicting the CVC in patients undergoing hemodialysis.

In the modeling cohort, receiver operating characteristic (ROC) curve analysis was performed to evaluate the discriminative ability of the nomogram. The model demonstrated good discrimination, with an area under the curve (AUC) of 0.789. indicating good discrimination (Figure 3). At the optimal cutoff value (−0.913), the sensitivity and specificity were 84.60% and 63.9%, respectively, indicating a favorable balance between identifying patients with CVC and excluding those without the condition. The calibration curve in the modeling cohort showed acceptable agreement between the predicted and observed probabilities of CVC. Although mild overestimation was observed in the higher predicted risk range, the bias-corrected curve obtained by bootstrap resampling generally followed the ideal line, with a mean absolute error of 0.054, indicating reasonable calibration of the model (Figure 4).

### External validation of the nomogram model

**Figure 5:**
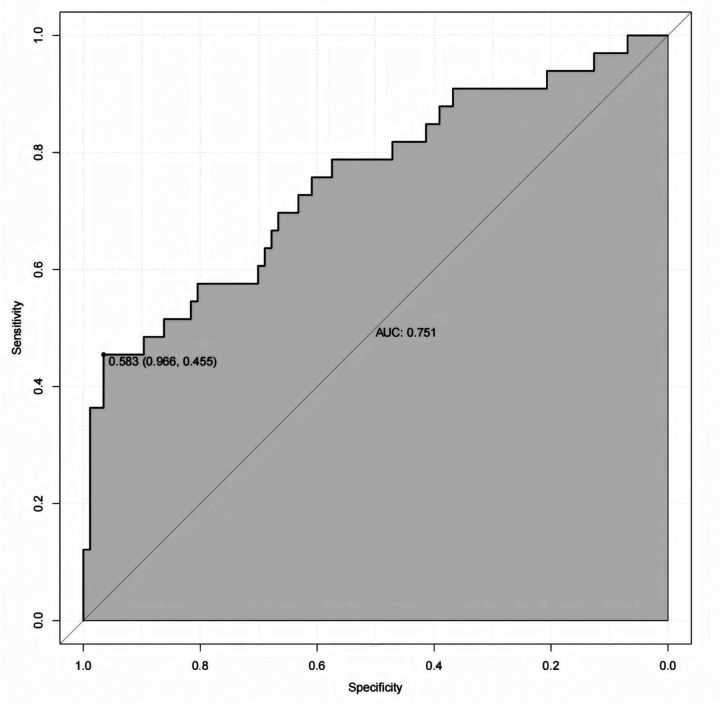
Receiver operating characteristic of the validation cohort for predicting the CVC in patients undergoing hemodialysis.

**Figure 6:**
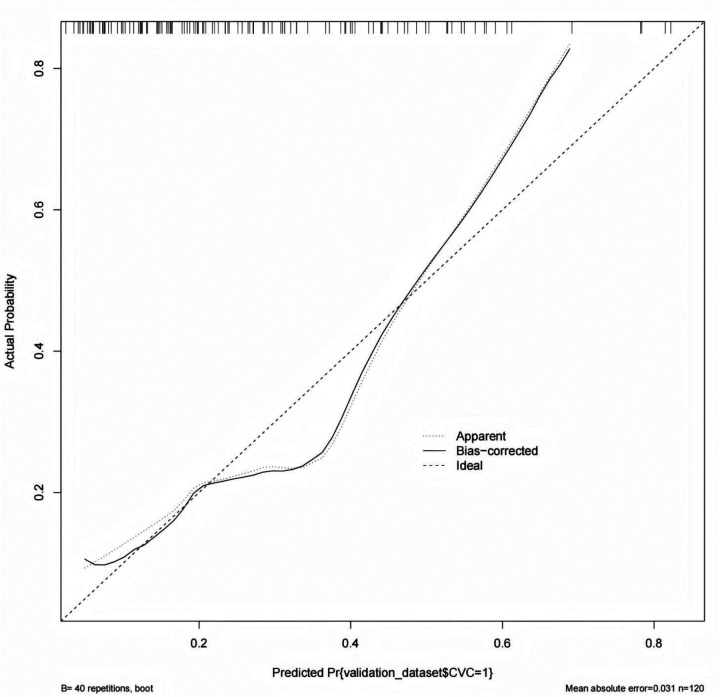
Calibration curve of the validation cohort for predicting the CVC in patients undergoing hemodialysis.

In the validation cohort, the nomogram maintained stable discriminatory performance, ability, yielding an an area under the curve (AUC) of 0.751 (Figure 5). The high specificity observed in this cohort (96.6%) indicates that the model is effective in correctly identifying patients without CVC, whereas the relatively low sensitivity (45.5%) suggests that some CVC events may not be detected. The calibration curve demonstrated good agreement between predicted and observed probabilities of CVC, indicating reliable predictive accuracy of the model in an independent dataset. The bias-corrected curve obtained by bootstrap resampling closely followed the ideal 45-degree line, with a low mean absolute error of 0.031, reflecting satisfactory calibration of the prediction model (Figure 6).

### Decision curve analysis (DCA) for clinical utility

**Figure 7:**
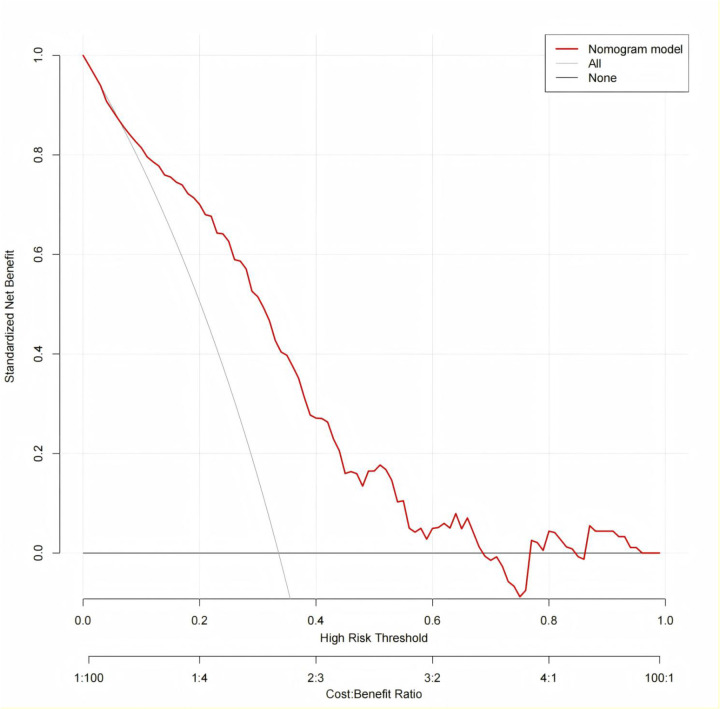
Decision curve analysis of nomogram for clinical utility in the internal validation.

**Figure 8:**
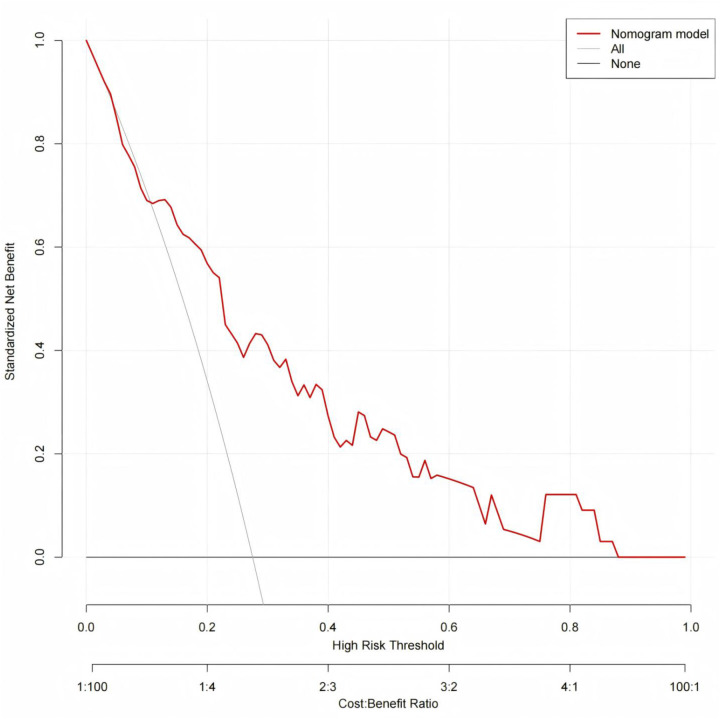
Decision curve analysis of nomogram for clinical utility in the external validation.

The clinical utility of the nomogram was assessed using decision curve analysis (DCA). In both the modeling and validation cohorts. the nomogram provided a higher net benefit than either the “treat-all” or “treat-none” strategies across a wide range of threshold probabilities (Figures 7, 8). This suggesting that the model has substantial clinical value for predicting the risk of CVC in patients undergoing maintenance hemodialysis.

## Discussion

Vascular calcification (VC) is characterized by the pathological deposition of calcium-phosphate complexes within the vascular wall, resulting in arterial stiffening and reduced vascular elasticity. As an important subtype of VC, cardiac valve calcification (CVC) has been shown in previous cross-sectional studies to be closely associated with systemic VC, and is recognized as a major contributor to morbidity and mortality among patients undergoing maintenance hemodialysis (14, 15).

In the present study, aortic valve calcification (AVC) was the most common form of CVC, affecting 75.40% of patients, followed by mitral valve calcification (MVC) (46.03%). In the modeling cohort, 33.58% of MHD patients developed CVC. The relatively lower prevalence observed in our study may be attributable to the younger age of the enrolled population and their shorter dialysis duration. In patients with chronic kidney disease (CKD), CVC predominantly involves the aortic and mitral valves, with reported prevalence ranging from approximately 25–64.8% (16), The wide variation across studies and centers likely reflects differences in the patient demographics, dialysis vintage, and comorbidity burden. Notably, CVC tends to develop 10–20 years earlier in patients with CKD than in the general population, and the annual incidence of AVC in MHD patients has been reported to be approximately 3.3%, increasing progressively with declining glomerular filtration rate (GFR) (17–19).

Multiple studies have demonstrated that CVC in MHD patients is associated with advanced age, comorbid diabetes mellitus, disturbances in calcium-phosphorus metabolism, chronic systemic microinflammatory, and conditions such as malnutrition or sarcopenia (2, 20–22). However, CVC is frequently underestimated or overlooked in routine clinical practice. Therefore, heightened clinical awareness and proactive surveillance of CVC, along with timely identification and management of modifiable risk factors, are essential to prevent or delay its progression. Accordingly, the development of an individualized risk prediction model for CVC in patients undergoing hemodialysis is of great clinical significance for guiding therapeutic decision-making and improving patient prognosis.

Bioelectrical impedance analysis (BIA) is an objective and noninvasive method for assessing body composition, nutritional status, and muscle mass. Routine application of BIA in clinical practice facilitates the evaluation of malnutrition, sarcopenia, and obesity-related risks, thereby providing additional prognostic information for the management of patients undergoing hemodialysis. However, few studies have investigated the association between body composition and CVC in maintenance hemodialysis patients. In the present study, BIA and handgrip strength were used to assess body composition and muscle function in order to explore their relationships with CVC in MHD patients. FTI reflects body fat distribution and represents a refined measure of body composition. In contrast to body mass index (BMI), which cannot accurately distinguish fat mass and lean mass, FTI provides a more precise classification of adiposity. Therefore, the use of FTI may facilitate more precise lipid- or nutrition-oriented management strategies at the level of body composition, thereby contributing to the prevention of vascular calcification.

Only the variable “with diabetes” was included in the statistical analysis, as some primary diseases occurred at very low frequencies, which could introduce bias. In addition, this variable overlapped with “cause of ESRD”, Therefore, simultaneous inclusion was avoided to prevent potential confounding. Multivariate logistic regression analysis demonstrated that age, male sex, dialysis duration ≥36 months, total cholesterol levels, and fat tissue index (FTI) were independent risk factors for CVC in patients undergoing hemodialysis. (1) Age. Cardiac valve calcification is a degenerative process that progresses over time. A meta-analysis (2), reported that CKD patients with CVC were older and exhibited lower ejection fractions and relatively impaired diastolic function. Cardiac valves are chronically exposed to abnormal mechanical stress, rendering hemodialysis patients more susceptible to calcific deposition. In the Multi-Ethnic Study of Atherosclerosis (MESA), the risk of aortic valve calcification (AVC) more than doubled per decade (risk ratio, 2.19; 95% *CI*, 1.84–2.61; *P* < 0.001) (23). (2) Male sex. Growing evidence indicates significant sex-related differences in valvular remodeling mechanisms, including distinct patterns of calcification and fibrosis (24). In women, valvular remodeling is characterized by increased fibrosis and relatively less calcification, whereas in men, calcification predominates and is more severe (25). These findings underscore the importance of sex-specific investigations to elucidate the underlying mechanisms and improve therapeutic strategies. Sex-related disparities in disease outcomes are thought to be largely attributable to differences in sex steroid hormone levels, particularly estrogen and testosterone. (3) Hemodialysis duration. With increasing dialysis duration, the cumulative effects of dialysis-related factors become more pronounced, primarily due to sustained systemic dysregulation, including chronic microinflammation, disturbances in calcium-phosphorus-parathyroid hormone metabolism, and persistent metabolic acidosis. Consistent with previous studies (26, 27), our results indicate that longer dialysis duration is a significant risk factor for CVC in patients undergoing hemodialysis. (4) Total cholesterol levels In the Framingham Offspring Study (FOS), levels of total cholesterol, which correlate highly with levels of low-density lipoprotein cholesterol (LDL-C), in early adulthood strongly predicted the presence of AVC later in life (adjusted OR per SD, 1.81; 95% *CI*, 1.55–2.11; *P* < 0.0001) (28). In the present study, elevated total cholesterol was identified as an independent risk factor for cardiac valve calcification in patients undergoing maintenance hemodialysis. Our findings provided indirect evidence that an elevated LDL-C levels may contribute to the development of CVC. This finding is biologically plausible, as lipid metabolism plays a crucial role in the pathogenesis of valvular calcification. Previous studies have demonstrated that low-density lipoprotein (LDL) cholesterol can infiltrate the valvular tissue, where it undergoes oxidative modification under conditions of enhanced oxidative stress and chronic inflammation commonly observed in CKD. Oxidized LDL promotes inflammatory responses and induces osteogenic differentiation of valvular interstitial cells through the activation of bone-related signaling pathways (29), thereby accelerating valvular calcification. Although LDL cholesterol was not separately analyzed in our study, total cholesterol largely reflects the circulating LDL burden and has been shown to be closely associated with valvular calcification in patients without CKD (30). (5) Fat tissue index. Although HGS was significantly lower in the CVC group than in the non-CVC group, multivariate logistic regression analysis did not reveal an independent association between HGS and CVC, indicating that further studies are required to clarify the relationship between muscle strength and CVC. Although HGS is a well-established marker of muscle function and frailty, it is more susceptible to short-term fluctuations related to hydration status, inflammation, and functional impairment in patients undergoing hemodialysis (31). Moreover, the lack of independent association between HGS and CVC in the multivariate model may be partially explained by the shared variance between muscle function, age, and nutritional status, suggesting that the effect of muscle strength on CVC may be indirect or mediated by other metabolic factors. Notably, our study is the first to demonstrate that increased FTI may serve as a robust predictor of CVC in hemodialysis patients. FTI represents the total amount of adipose tissue, comprising both pure fat and its associated water content. Previous studies (32) have shown that loss of lean tissue index (LTI) and gain of FTI are independently associated with all-cause mortality, regardless of demographic characteristics, laboratory parameters, and comorbidities. Additionally, Wang et al. (16) reported that increased visceral fat area may act as a potential marker for CVC in MHD patients, supporting a link between adipose tissue and vascular diseases. The mechanisms underlying the association between increased FTI and CVC in MHD patients remains unclear. Adipose tissue functions as a major endocrine organ with diverse physiological roles (33). It secretes a variety of peptides and metabolites, collectively termed adipokines, which regulate perivascular inflammation, promote osteogenic differentiation, proliferation, and apoptosis of vascular cells; and induce ectopic calcium-phosphorus deposition, thereby contributing to vascular calcification (34, 35).

Our study did not identify a significant association between serum calcium or phosphorus levels and CVC in MHD patients. This inconsistency may be partly attributable to widespread use of calcium carbonate and various phosphate binders at our center in recent years, which may have influenced the serum calcium and phosphorus concentrations. In addition, patients were routinely monitored and managed according to current clinical guidelines, including standardized pharmacological treatment for disorders of mineral metabolism such as secondary hyperparathyroidism, which may have further attenuated the variability of serum calcium and phosphorus levels. As a result, the impact of these parameters on CVC may have been underestimated in the present analysis. Further studies are warranted to verify these finding in hemodialysis populations. Moreover, given the cross-sectional nature of this study rather than a prospective cohort design, no direct causal relationship between these biochemical parameters and CVC can be inferred.

Nomogram models based on core diagnostic indicators are useful tools for individualized risk assessment and early disease identification. In the present study, after integrating significant predictors identified through multivariate analysis, the constructed visual nomogram demonstrated an area under the curve (AUC) exceeding 0.75 in both internal and external validation, indicating good discrimination and calibration. Decision curve analysis (DCA) further suggested that the nomogram has substantial clinical utility in both validation cohorts. Using this model, clinicians can estimate the risk of CVC in patients undergoing hemodialysis by summing the corresponding risk factor scores, and thus, thereby facilitating early identification and prevention of high-risk CVC progression.

Several limitations of this study should be acknowledged. First, this was a single-center, cross-sectional study with a relatively small sample size, which may limit the generalizability of the findings. Moreover, cross-sectional designs are inherently limited for the development of predictive models, as they preclude assessment of temporal relationships and dynamic changes over time. Although internal and external validation were performed, the number of patients included in the external validation cohort was relatively small, which may have affected the stability and robustness of the validation results. Therefore, further validation in larger, multicenter, and preferably prospective cohorts is required to confirm the reliability and clinical utility of the nomogram model. In addition, the lack of information on the duration of chronic kidney disease prior to dialysis initiation represents another limitation, as prolonged exposure to uremic and mineral metabolic disturbances before dialysis may contribute to the development of valvular calcification. Although LDL cholesterol may represent a more direct atherogenic factor, it was not included in the nomogram due to incomplete availability and its strong correlation with total cholesterol, which may limit further refinement of lipid-related risk stratification. Future studies incorporating a broader range of clinical and biochemical variables may further improve the predictive accuracy and clinical applicability of the model.

In conclusion, patients undergoing hemodialysis who are older, male, have a dialysis duration ≥ 36 months, elevated total cholesterol levels, and increased fat tissue index are at higher risk of developing CVC. A nomogram prediction model based on these risk factors may serve as a practical tool in clinical settings to reasonably assess CVC risk and implement targeted preventive strategies to reduce its incidence. Clinically, integrating patients' lipid profiles with body composition analysis is essential for individualized nutritional management, which may play a critical role in preventing cardiac valve calcification and further reducing the burden of cardiovascular disease.

## Data Availability

The datasets used during the current study available on reasonable request. Requests to access these datasets should be directed to 1606454802@qq.com.
